# An Assessment of Clinical Research Self-Efficacy among Researchers at the Largest Healthcare Institute in Qatar: Recommendations and Future Actions

**DOI:** 10.1177/23821205241233425

**Published:** 2024-06-07

**Authors:** Seba Qussini, Saad Shahbal, Ross MacDonald, Samer Hammoudeh, Zeina Al-Ghoul, Kris Diericks

**Affiliations:** 1 The Medical Research Center, 36977 Hamad Medical Corporation, Doha, Qatar; 2 Faculty of Medicine, Centre for Biomedical Ethics and Law, 26657 KU Leuven, Leuven, Belgium; 3 Department of Medicine, 36977 Hamad Medical Corporation, Doha, Qatar; 4 Distributed eLibrary, 36579 Weill Cornell Medicine—Qatar, Education City, Doha, Qatar; 5 Faculty of Medicine, 52946 Bahcesehir University, Istanbul, Turkey

**Keywords:** clinical research, self-efficacy, training, research integrity, continuing education, Qatar

## Abstract

**OBJECTIVES:**

Clinical research professionals must be equipped with adequate training in sound scientific methods and appropriate ethics. In this study, we aimed to assess the current clinical research self-efficacy of researchers at Hamad Medical Corporation (HMC). We also evaluated the effects of training courses on researchers’ self-efficacy.

**METHODS:**

Utilizing a cross-sectional design, we used the shortened Clinical Research Appraisal Inventory (CRAI-12) through an online survey to assess the current clinical research self-efficacy of 600 researchers at HMC, Doha, Qatar. After conducting descriptive analyses, unpaired *t* test and ANOVA were used to determine significant mean percentages between variables. Pearson correlation coefficients were also calculated to measure the association among the interval variables. All tests were 2-sided, and significance was defined as *P* < .05.

**RESULTS:**

For all questions, except those related to “funding,” most participants scored on the upper half of the scale (>5), reflecting higher self-efficacy for the topics covered in CRAI. Gender differences were significant across all factors, with males reporting higher levels of self-assessed efficacy and in clinical research. Other factors such as higher education degrees and previous (external) clinical research training were also associated with higher self-reported clinical research efficacy.

**CONCLUSIONS:**

The findings of this study indicate that researchers at HMC possess high clinical research self-efficacy overall, but lower self-efficacy in securing funding. Gender and education level positively influence self-efficacy across CRAI factors. Notably, clinical research training boosts self-efficacy, especially when obtained outside HMC. In conclusion, healthcare providers are strongly encouraged to engage in effective clinical research training courses, both within and outside of their healthcare institutions, to improve their clinical research efficacy and enhance clinical practice.

## Introduction

The importance of research integrity is paramount in clinical research, especially that involves direct contact with human subjects.^1^ Numerous issues have recently threatened the reliability and integrity of clinical research findings, including methodological flaws, reproducibility concerns, selective reporting, and ethical issues.^2^ Hence, maintaining the credibility of research results hinges on the cultivation of a competent workforce, well-versed in robust scientific methodologies and ethical training.^1,3^ The COVID-19 outbreak highlighted the necessity of swiftly applying research outcomes to improve healthcare practices. Consequently, it becomes imperative to regularly assess the clinical research abilities and proficiency of healthcare professionals, ensuring their readiness to promptly address any urgent challenges requiring clinical research expertise.^4^

A preliminary step to identifying current barriers and needs in the development of clinical researchers’ career trajectories, and to ultimately designing appropriate interventions, is the assessment of self-efficacy.^5^ Self-efficacy pertains to an individual's assessment of their capacity to learn or execute tasks at specified levels of proficiency.^6^ Tools that assess self-efficacy stem from the theory that if one is more confident about completing a task successfully, then one is more likely to do so.^5,7^ The underpinning theory of self-efficacy tools is also linked to the social cognitive career theory which relates higher confidence in performing certain tasks to more plausible chances in pursuing a career in that field.^8^ In clinical research, self-efficacy tools employ self-assessed confidence as a factor influencing clinical research productivity. They gauge an individual's perceived confidence in successfully executing a research-related task and correlate this estimation with the likelihood of both completing the task successfully and pursuing a career related to clinical research.^5^ Thus, clinical research self-efficacy tools are an important construct in researchers’ career assessment, they can predict students’ interest in pursuing a career in clinical research and researchers’ actual performance and productivity.^9^ While self-efficacy is widely acknowledged as a positive predictor of scholarly productivity and research interest,^10,11^ it is important to note that self-efficacy should not be perceived as the sole determinant of researchers’ clinical research competencies^7^ and to acknowledge the presence of other factors that might disrupt the relation between self-efficacy and actual performance.^11^ These factors include task complexity, work environment conditions, and lack of continuing professional development courses and training.^10^ To address these factors, several interventions have been suggested including efficacy-enhancement training, comprehensive task-related instructions and job specifications, use of clear key performance indicators, providing necessary technological means and offering performance-related incentives.^6,10^

Various tools are available to measure research self-efficacy such as Research Self-Efficacy Scale (RSES),^12^ Self-Efficacy in Research Measure,^13^ and Clinical Research Appraisal Inventory (CRAI).^5^ These tools ask respondents to evaluate their confidence to successfully undertake research-related tasks. Although these tools are developed from different content domains and cater to diverse populations (students, graduate students, and researchers), they are grounded in the same theoretical frameworks: Bandura's self-efficacy theory^7^ and social cognitive career theory.^14^

The CRAI developed by Mullikin et al^5^ is a self-efficacy tool comprised 92 items that aims to evaluate researchers’ confidence in performing clinical research tasks through 10 elements: study design; planning and managing a research study; funding a study; collaborating with others; conceptualizing a study; protecting research subjects and the responsible conduct of research; collecting, recording, and analyzing data; interpreting data; reporting a study and presenting a study. While the CRAI is reliable (Cronbach's alpha = 0.96), a 92-item tool is perceived to be extremely cumbersome to use. Hence different variations of the CRAI have been derived and used in different studies. Lipira et al^15^ used a 76-item version of the CRAI to investigate clinical research self-efficacy among scholars at Washington University School of Medicine after participation in research training programs. The authors observed a significant increase in clinical research self-efficacy 1 year after clinical research training,^15^ which supports the hypothesis that participation in a research training program can contribute to the development of clinical research self-efficacy and eventually translate into better research outcomes.^16,17^ Robinson et al condensed the 92-item tool into a smaller version of 12 items, the CRAI-12.^18^ According to a recent study,^19^ The implementation of a 1-year training program resulted in a remarkable 30% increase in CRAI-12 scores, with clinical research self-efficacy increasing from a primary mean score of 6.0/10 to 7.9/10.

Due to its ability to identify self-perceived strengths and weaknesses, clinical research self-efficacy can facilitate the development of needed interventions and research training programs.^9^ In addition to their role in developing training courses, clinical research self-efficacy tools also act as checkpoints to verify that current researchers possess the necessary knowledge to fulfill their assigned responsibilities efficiently.^1^ Notably, a common factor contributing to research misconduct is the lack of clinical research knowledge and expertise.^2^ Hence, identifying commonly perceived weaknesses among researchers and implementing tailored training programs within continuing education programs stands as a preventive measure to promote responsible research conduct.^20^ Many efforts and initiatives have been undertaken globally to develop specialized training courses that effectively uphold the integrity of clinical research.^1^ In this vein, Hamad Medical Corporation (HMC) made considerable efforts to educate and train its faculty and researchers. Such efforts include routine research training workshops (4-day training course on literature review, research methodology, statistical analysis, research ethics, and publication) and collaborating with renowned educational and research institutes such as Harvard T. H. Chan School of Public Health and The *British Medical Journal*.

Previous studies have highlighted that self-efficacy is a dynamic construct that changes over time^21^ and can be affected with multiple factors such as gender,^22^ level of education,^23^ clinical experience,^24^ and received training.^15^ Consequently, identifying factors to design suitable interventions can indeed improve clinical research self-efficacy. In this regard, evaluating researchers’ self-efficacy in Qatar is crucial for customizing specialized training programs that aims to uphold ethical standards and ensure the scientifically robust execution of clinical research. While few previous studies have investigated aspects of research self-efficacy among researchers in Qatar,^16^ a noticeable gap exists in understanding the specific determinants of research self-efficacy among researchers, as the regional body of evidence focuses on self-efficacy among students.^16^ Therefore, this study seeks to bridge this gap by evaluating the current self-efficacy of the clinical research workforce in HMC, as one of the leading research institutions in Qatar, and investigating the influence of research training courses, demographic variables, and professional factors on the reported research self-efficacy among researchers at HMC. The primary research question is “what is the level of research self-efficacy among researchers in HMC?” while the secondary questions are “what are the determinants of research self-efficacy among researchers in HMC?” and “how does research training, whether obtained internally or externally, affect HMC researchers’ self-efficacy?”

## Materials and methods

### Study design

This is a cross-sectional study, utilizing a web-based anonymous questionnaire created in SurveyMonkey (https://www.surveymonkey.com/) that was sent out to applicants (and their teams) who had submitted research proposals to HMC-MRC (Medical Research Center) in 2021. Data were gathered during 1 month (May 24, 2022-June 23, 2022). Two reminders were sent out to all participants, irrespective of received responses, to increase the response rate. The initial reminder was sent out 10 days after the initial invitation, which was followed by a second reminder 10 days later.

### Sampling technique and sample size calculation

Participants were contacted through email with a link to the survey in SurveyMonkey. Sample size was calculated considering a 50% self-efficacy and a 5% error rate with 95% confidence interval, hence, a maximum of 400 randomly selected participants would be needed in this study based on the below formula:

Sample size *n *= [DEFF × N*P*(1 − *P*)]/[(*d*^2^/*z*^2^_1−α/2_ × (N − 1) + *P* × (1 − *P*)]

where *P* = .50, N = 10,000, *d* = 5%, *z* = 1.96. Design effect = 1.2 and non-response rate = 35%.

Results from OpenEpi, version 3, open-source calculator—SSPropor^25^

Regarding sampling technique in the survey, a design effect of 1.2 was utilized to eliminate the cluster effect in the sample.^25^ Therefore, 600 participants were eventually contacted in this study. These were HMC-affiliated physicians and researchers who have conducted research in HMC in the past and are registered on the HMC online research portal ABHATH.

### Study setting and population

The study was conducted at the HMC-MRC in Doha, Qatar. HMC is the main healthcare provider in Qatar. It is a network of multiple specialized hospitals and service centers. The MRC at HMC is the corporate research department responsible for the research governance, funding, and administration across HMC. To achieve the targeted sample size of 600 HMC researchers, 178 approved research proposals were extracted from a total of 1100 submitted during 2021. On average, each proposal included 3.4 investigators in the core team. Research coordinators and research assistants were not included in this study, as more suitable tools are available to assess self-efficacy among such research support team members, such as Competency Index for Clinical Research Professionals (CICRP).^26^ Unlike the CRAI, CICRP is designed to assess self-efficacy among research coordinators in performing certain clinical research-related tasks.^23,26^ Biostatisticians were also excluded as they are usually not directly involved in the areas that the CRAI tool explores. The findings of this study are reported according to CHERRIES (Checklist for Reporting Results of Internet E-Surveys) checklist of Internet E-surveys.^27^

### Tool

**Figure 1.**
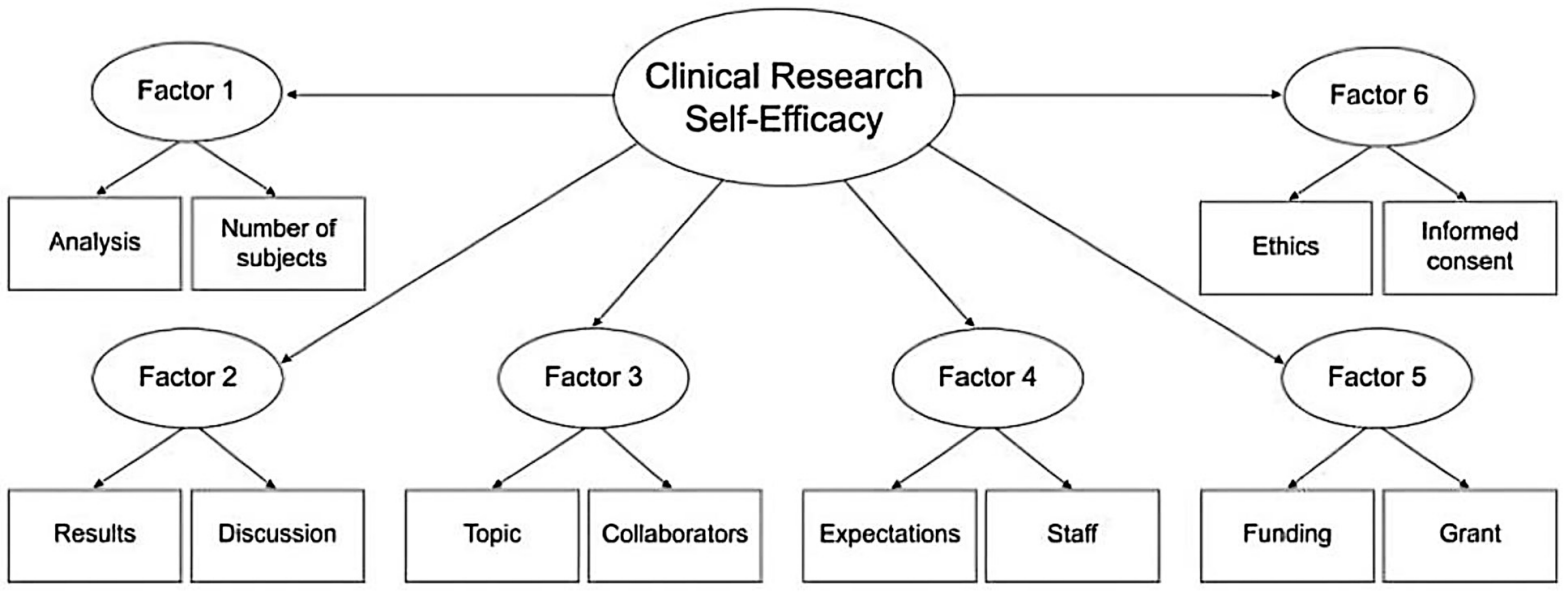
The factor model for the shortened CRAI-12 as described by Robinson et al.^18^ The tool's factors and items are described in full in Table 2.Abbreviation: CRAI, Clinical Research Appraisal Inventory.

The original tool, developed by Mullikin et al, encompassed 92 items across 10 subscales, detailed in the Introduction. The tool demonstrated robust reliability as indicated by a median coefficient alpha of 0.96 across the 10 subscales and a median test-retest reliability of 0.88.^5^ However, due to its length, a more concise version was created. The shortened CRAI-12 comprises 6 factors and 12 items in total (Figure 1) with an internal consistency similar to that of Mullikin's original tool (Cronbach's alpha ranged from 0.80 to 0.94). Additionally, regarding internal validity, both tools exhibited adequate validity, supported by convergent factor validity of 0.27 (*P* < .01) for both tools and divergent factor validity of 0.08 (*P* = .32) and 0.10 (*P* = .23) for the CRAI-92 and CRAI-12, respectively. In a similar fashion to the original tool, each item in this tool can be scored on an 11-point scale, where 0 reflects no self-confidence in a clinical research-related skill and 10 reflects total self-confidence. The shortened version was used in the current study to engage more researchers, given the shorter response time required.

### Informed consent and ethical considerations

The study was approved by HMC institutional review board (Ref No: MRC-01-22-050) and conducted in full conformance with principles of the Declaration of Helsinki,^28^ good clinical practice^29^ and within the laws and regulations of the Ministry of Public Health in Qatar.^30^ Emails inviting participation included a summary of the study and an electronic link to the survey. The requirement for consent was waived by HMC IRB and substituted with an information sheet. The information sheet was attached to the survey inviting email, briefing the invitees on its purpose, voluntary participation, and assuring anonymity of the collected responses. Participants were not offered any incentive in exchange for their responses.

### Data collection and management

Data collection for this study was conducted exclusively in the English language since it is the official language of communication within the corporation along with Arabic. HMC researchers, the target population for this survey, typically submit their proposals in English. Hence, it was assumed that they possessed the necessary proficiency to effectively respond to the survey in English. The survey consisted of a total of 19 items: 7 demographic/profession-related questions (Table 1) and 12 items from the CRAI-12 tool (Table 2). The survey was designed in a single-page format, requiring participants to scroll down to view and respond to all the items. The average completion time for the survey (as calculated by the program) was estimated to be approximately 4 minutes, whereas the actual average completion time for all participants was 5 minutes and 37 seconds, with the shortest completion time recorded at 2 minutes and 9 seconds. Data were captured electronically through the SurveyMonkey app and imported into the authors’ account and stored in a secured server location accessible only to the study team. Since no personal identifiers were collected from the respondents, serial numbers were generated for each data set by the survey app.

**Table 1.** Sociodemographic and professional factors among the survey respondents.

VARIABLE	CATEGORY	N (%)
Gender (n = 80)	Male	55 (68.8)
	Female	25 (31.2)
Nationality (n = 80)	Asian	43 (53.8)
	African	22 (27.5)
	European	9 (11.3)
	Americas	6 (7.5)
Seniority of position (n = 80)	Resident	5 (6.3)
	Fellow	4 (5.0)
	Specialist	6 (7.5)
	Associate consultant	5 (6.3)
	Consultant	10 (12.5)
	Senior consultant	13 (16.3)
	Others	37 (46.3)
Education (n = 80)	Bachelor’s	12 (15)
	MSc	18 (22.5)
	MD	25 (31.3)
	PhD	25 (31.3)
Department (n = 80)	Medicine	21 (26.3)
	Surgery	10 (12.5)
	Pharmacy	6 (7.5)
	Nursing	9 (11.3)
	Others	34 (42.5)
Training in HMC (n = 80)	No	39 (48.8)
	Yes	41 (51.2)
Training outside HMC (n = 80)	No	38 (47.5)
	Yes	42 (52.5)

Abbreviation: HMC, Hamad Medical Corporation.

### Statistical analysis

The collected data set was transferred for statistical analysis to IBM SPSS, version 22.0 (IBM, Armonk, NY). Descriptive statistics in the form of mean percentage and standard deviations were calculated for continuous variables (such as self-efficacy value), whereas frequency and percentages were calculated for categorical variables (such as professional designation and specialty). The mean percentage was calculated by taking the average of the relevant items and multiplying by 100. An unpaired student *t* test was used to determine significant mean percentage differences in the following interval factors: designing and collecting; reporting, interpreting, and presenting; conceptualizing and collaborating; planning; funding and protecting; gender; training outside and inside HMC. A 1-way analysis of variance (ANOVA) was also performed according to other sociodemographic variables for these interval variables. Pearson correlation coefficients were calculated to measure the association among the interval variables. All tests were 2-sided and significance was defined as *P* < .05.

## Results

We received 80 responses from the invited sample and the sociodemographic and professional factors of the participants who completed our survey are portrayed in Table 1. Most respondents were males and of Asian nationalities, holding positions in allied health professions such as nursing or pharmacy. Furthermore, slightly over half of the participants had received clinical research training, either within or outside of HMC.

### Research question 1

Question 1 pertains to the level of clinical research self-efficacy of HMC researchers. Table 2 represents the reported research self-efficacy percentage mean scores for respondents. The results showed adequate research self-efficacy among participants as most scores were on the upper half of the scale. This is consistent with previous studies utilizing the CRAI tool, where participants’ scores were within the upper half of the tool as well.^31–33^ For instance, Bierer et al reported the following means and standard deviation for the clinical research appraisal inventory-short form: conceptualizing a study 7.2 (1.3), study design and analysis 5.9 (1.7), collaborating with others 7.7 (1.3), organizing a study 7.4 (1.8), responsible research conduct 3.6 (2.8) and reporting a study 7.1 (1.5).

**Table 2.** The percentage mean scores of HMC researchers’ self-reported clinical research efficacy using CRAI-12 (80 responses). The CRAI-12 tool has a 6-factor structure with 2 items listed (all items ranged from 0 to 10) within each factor.

CRAI-12 ITEM	PERCENTAGE MEAN ± SD (AVERAGE LIKERT SCORE)
Factor 1: Designing and collecting	65.4 ± 22.5 (6.5)
Q1: Design the best data analysis strategy for your study (analysis)	63.9 ± 22.8 (6.4)
Q2: Determine an adequate number of subjects for your research project (number of subjects)	66.9 ± 24 (6.7)
Factor 2: Reporting, interpreting, and presenting	74.1 ± 22.8 (7.4)
Q3: Write the results section of a research paper that clearly summarizes and describes the results, free of interpretative comments (results)	74.0 ± 23.0 (7.4)
Q4: Write a discussion section for a research paper that articulates the importance of your findings relative to other studies in the field (discussion)	77.3 ± 24.0 (7.7)
Factor 3: Conceptualizing and collaborating	74.7 ± 19.8 (7.5)
Q5: Select a suitable topic area for study (topic)	79.0 ± 19.3 (7.9)
Q6: Identify faculty collaborators from within and outside the discipline who can offer guidance to the project (collaborators)	70.4 ± 23.7 (7.0)
Factor 4: Planning	66.9 ± 22.1(6.7)
Q7: Set expectations and communicate them to project staff (expectations)	73.6 ± 21.7 (7.4)
Q8: Ask staff to leave the project team when necessary (staff)	60.1 ± 28.2 (6.0)
Factor 5: Funding	59.1 ± 26.5 (5.9)
Q9: Describe the proposal review and award process for a major funding agency, such as the National Institutes of Health, National Science Foundation, or other foundation (funding)	57.0 ± 29.7 (5.7)
Q10: Locate appropriate forms for a grant application (grant)	61.1 ± 26.8 (6.1)
Factor 6: Protecting	72.3 ± 23.8 (7.2)
Q11: Describe ethical concerns with the use of placebos in clinical research (ethics)	66.5 ± 27.9 (6.7)
Q12: Apply the appropriate process for obtaining informed consent from research subjects (informed consent)	78.1 ± 23.1 (7.8)

Abbreviations: HMC, Hamad Medical Corporation; CRAI, Clinical Research Appraisal Inventory.

### Research question 2

Research question 2 concerns the determinants of clinical research efficacy among researchers in HMC. The outcomes for factors examined using *t* tests are displayed in Table 3, while Table 4 illustrates the results for factors analyzed through ANOVA. The results show that gender and level of education were found to be significant across all CRAI factors. While seniority of position exhibited a noteworthy trend with statistically significant correlation for all factors except factors 1 and 2. Other factors such as nationality and specialty did not predict the outcome (clinical research self-efficacy). Surgeons reported the highest percentage mean self-efficacy for all CRAI factors.

**Table 3.** Association of gender and received training with mean percentage research self-efficacy across the 6 factors of the CRAI-12 tool.

		FACTOR 1: DESIGNING AND COLLECTING (PERCENTAGE MEAN ± SD)	FACTOR 2: REPORTING, INTERPRETING, AND PRESENTING (PERCENTAGE MEAN ± SD)	FACTOR 3: CONCEPTUALIZING AND COLLABORATING (PERCENTAGE MEAN ± SD)	FACTOR 4: PLANNING (PERCENTAGE MEAN ± SD)	FACTOR 5: FUNDING (PERCENTAGE MEAN ± SD)	FACTOR 6: PROTECTING (PERCENTAGE MEAN ± SD)
Gender	Male Mean ± SD	70.3 ± 20.4	81.2 ± 16.7	81.1 ± 14.8	72.6 ± 17.2	64.5 ± 24.5	76.9 ± 21.8
	Female Mean ± SD	54.6 ± 23.6	58.6 ± 26.9	60.6 ± 22.3	54.2 ± 26.3	47 ± 27.4	62.2 ± 25.3
	*T*-value (f)	3.03 (78)	4.6 (78)	4.9 (78)	3.7 (78)	2.9 (78)	2.7 (78)
	*P*-value	0.003	0.001	0.001	0.001	0.005	0.01
	95% CI of difference	[5.4 to 26.0]	[12.8 to 32.4]	[12.1 to 28.9]	[8.6 to 28.3]	[5.3 to 29.7]	[3.7 to 25.7]
Research training in HMC	Yes Mean ± SD	64.9 ± 20.9	75.6 ± 21.9	76.7 ± 20.5	66.5 ± 22.5	57.3 ± 28.4	71.2 ± 25.6
	No Mean ± SD	65.9 ± 24.4	72.6 ± 23.9	72.6 ± 19.2	67.3 ± 21.9	60.9 ± 24.7	73.5 ± 22.1
	*T*-value (f)	0.20 (78)	−0.59 (78)	−0.93 (78)	0.17 (78)	0.60 (78)	0.42 (78)
	*P*-value	0.84	0.56	0.35	0.87	0.55	0.68
	95% CI of difference	[−9.1 to 11.1]	[−13.3 to 7.2]	[−12.9 to 4.7]	[−9.0 to 10.7]	[−8.3 to 15.4]	[−8.4 to 12.9]
Research training outside HMC	Yes Mean ± SD	72.6 ± 18.6	83.2 ± 15.9	81.2 ± 14.5	70.4 ± 18.7	63.1 ± 27.4	76.5 ± 20.9
	No Mean ± SD	57.4 ± 23.9	64.1 ± 25.3	67.1 ± 22.2	63.0 ± 24.9	54.6 ± 25.2	67.6 ± 26.2
	*T*-value (f)	−3.2 (78)	−4.1 (78)	−3.5 (78)	−1.5 (78)	−1.4 (78)	−1.7 (78)
	*P*-value	0.002	0.001	0.001	0.14	0.15	0.09
	95% CI of difference	[−24.8 to −5.7]	[−28.4 to −5.6]	[−22.7 to −6.2]	[−17.1 to 2.4]	[−20.2 to 3.3]	[−19.4 to 1.6]

Abbreviations: CRAI, Clinical Research Appraisal Inventory; CI = confidence interval.

**Table 4.** Association of sociodemographic and professional factors with mean percentage research self-efficacy across the 6 factors of the CRAI-12 tool.

		FACTOR 1: DESIGNING AND COLLECTING (PERCENTAGE MEAN ± SD)	FACTOR 2: REPORTING, INTERPRETING, AND PRESENTING (PERCENTAGE MEAN ± SD)	FACTOR 3: CONCEPTUALIZING AND COLLABORATING (PERCENTAGE MEAN ± SD)	FACTOR 4: PLANNING (PERCENTAGE MEAN ± SD)	FACTOR 5: FUNDING (PERCENTAGE MEAN ± SD)	FACTOR 6: PROTECTING (PERCENTAGE MEAN ± SD)
Education	Bachelor’s	55.5 ± 29.3	49.2 ± 27.1	60.8 ± 25.9	53.3 ± 28.4	42.9 ± 29.7	62.9 ± 26.2
	Master’s	54.4 ± 26.3	65.8 ± 24.1	63.1 ± 18.4	55.3 ± 23.2	51.7 ± 29.1	60.3 ± 25.2
	MD	67.0 ± 14.5	78.8 ± 14.3	80 ± 14.6	72.8 ± 16.5	54.8 ± 22.8	73.4 ± 23.6
	PhD	76.6 ± 17.3	87.4 ± 14	84.4 ± 14.4	75.8 ± 16.3	76.4 ± 17.1	84.4 ± 15.4
	*F*-ratio (df)	5.0 (3)	12.6 (3)	8.5 (3)	6.1 (3)	7 (3)	4.9 (3)
	*P*-value	0.003	0.001	0.001	0.001	0.001	0.003
Nationality	Asian	68.0 ± 22.0	73.9 ± 24.1	75.2 ± 19.9	65.6 ± 23.9	59.5 ± 25.5	73.9 ± 23.2
	African	57.9 ± 21.4	70.5 ± 19.3	73.2 ± 18.3	67.0 ± 18.3	51.5 ± 28.8	64.7 ± 23.3
	European	69.4 ± 17.4	86.1 ± 16.9	79.4 ± 13.1	74.4 ± 9.8	71.7 ± 18.9	83.9 ± 13.9
	Americans	67.5 ± 34.7	70.8 ± 32.2	69.2 ± 32.8	64.2 ± 34.6	64.2 ± 32.2	70.8 ± 36.4
	*F*-ratio (df)	1.1 (3)	1.1 (3)	0.4 (3)	0.4 (3)	1.4 (3)	1.5 (3)
	*P*-value	0.35	0.37	0.77	0.74	0.26	0.21
Seniority	Resident	40 ± 27.2	49 ± 26.1	45 ± 26.9	34 ± 21.6	26 ± 18.5	31 ± 29.7
	Fellow	63.8 ± 19.3	77.5 ± 8.7	82.5 ± 13.2	80 ± 10.8	51.3 ± 29.5	62.5 ± 32
	Specialist	74.2 ± 15.9	80.8 ± 12.4	86.7 ± 14	75 ± 21.9	55 ± 34.8	85.8 ± 13.2
	Associate consultant	54 ± 32.3	73 ± 30.1	71 ± 27	58 ± 27.7	50 ± 28.1	59 ± 33.6
	Consultant	72 ± 15.1	81 ± 15.1	76 ± 16.6	72 ± 25.1	72.5 ± 15.7	81.5 ± 13.3
	Senior consultant	71.2 ± 17.9	85 ± 14.3	86.9 ± 11.6	78.8 ± 11.4	73.1 ± 17.9	83.1 ± 12.3
	Others	65.3 ± 23.3	70.5 ± 25.5	71.8 ± 18	64.2 ± 19.9	57.7 ± 56.8	72.3 ± 21.1
	*F*-ratio (df)	1.8 (6)	2.1 (6)	4.1 (6)	3.9 (6)	2.9 (6)	5.1 (6)
	*P*-value	0.11	0.07	0.001	0.002	0.01	0.001
Specialty	Medicine	66.2 ± 23.9	80.7 ± 22.9	79.5 ± 22.1	70.3 ± 22.8	57.1 ± 32.3	73.3 ± 26.6
	Surgery	68 ± 14.6	83.5 ± 12	80 ± 16.5	74 ± 20.5	69 ± 10.7	79 ± 9.4
	Pharmacy	55 ± 21.9	71.7 ± 21.6	75.8 ± 19.1	68.3 ± 12.1	53.3 ± 36.3	65.8 ± 26.2
	Nursing	63.9 ± 31.4	63.3 ± 26.3	66.7 ± 20.9	64.4 ± 27.2	57.2 ± 21.8	75.6 ± 20.7
	Others	66.3 ± 21.8	70.6 ± 23.5	72.1 ± 18.9	63.1 ± 22.2	58.8 ± 25.9	70 ± 25.8
	*F*-ratio (df)	0.4 (4)	1.6 (4)	1 (4)	0.7 (4)	0.4 (4)	0.4 (4)
	*P*-value	0.83	0.18	0.40	0.63	0.78	0.79

Abbreviation: CRAI, Clinical Research Appraisal Inventory.

### Research question 3

Question 3 delves into the effects of training on clinical research self-efficacy as analyzed statistically through *t* test (Table 3). In general, participants who received training outside HMC reported higher clinical research self-efficacy than participants who did not receive external training, with statistical significance achieved through the first 3 CRAI factors. However, this effect was not as pronounced for participants who received internal (inside HMC) clinical research training, as there was no significant difference in the reported mean self-efficacy for all CRAI factors between participants who received and those who did not receive clinical research training in HMC.

### Secondary analysis

There was a significant positive correlation (*P* = .001) between all CRAI factors, such that higher reported mean self-efficacy for 1 factor was directly proportional to the reported mean self-efficacy of other factors (Table 5). However, there was no significant correlation between any of the CRAI factors and the number of years in HMC indicating that seniority by employment duration was not relevant to participants’ research self-efficacy (Table 5).

**Table 5.** Correlation between CRAI factors and years in HMC.

Variable	Factor 1 (*r*)	Factor 2 (*r*)	Factor 3 (*r*)	Factor 4 (*r*)	Factor 5 (*r*)	Factor 6 (*r*)
Factor 2 (*r*)	0.75*	—	—	—	—	—
Factor 3 (*r*)	0.69*	0.77*	—	—	—	—
Factor 4 (*r*)	0.68*	0.69*	0.76*	—	—	—
Factor 5 (*r*)	0.63*	0.59*	0.64*	0.69*	—	—
Factor 6 (*r*)	0.65*	0.59*	0.72*	0.62*	0.71*	—
Years in HMC (*r*)	−0.05	−0.01	0.0	0.03	−0.04	−0.02

Abbreviations: CRAI, Clinical Research Appraisal Inventory; HMC, Hamad Medical Corporation.

*P < .05.

## Discussion

This study employed the CRAI-12 tool to assess research self-efficacy and examine the impact of training on clinical researchers at HMC, the primary provider of secondary and tertiary healthcare in Doha, Qatar. Additionally, we explored demographic and professional factors influencing research self-efficacy. When considering all questions, most participants scored more than 50%, reflecting higher self-efficacy of the topics covered under this tool. The lowest percentage mean score among the 6 factors of clinical research competencies revolved around questions that assess self-efficacy in obtaining research funds (factor 5). Conversely, the percentage mean scores were the highest for Factors 2 and 3; “reporting, interpreting and presenting” and “conceptualizing and collaborating,” at about 74%.

We recognize that the current sample size might have affected the statistical significance of any of the measured variables. However, we found gender, seniority of position, educational level, and exposure to external training (outside of HMC) to have influenced research self-efficacy in the study sample. In contrast, nationality, departmental affiliation, and length of employment appeared to have no significant impact. Respondents overall reported prominent levels of clinical research self-efficacy, however, the standard deviation for all percentage mean scores was large, indicating that levels of confidence in performing research tasks varied widely.

Females reported lower levels of research self-efficacy when compared to males across all factors. Males reported the highest self-efficacy for Factor 2 “reporting, interpreting and presenting” and Factor 3 “conceptualizing and collaborating” at 81% compared to females who reported 58.6 ± 26.9 (*P* = .001) and 60.6 ± 22.3 (*P* = .001), respectively, for the same factors. Among all scores for females, the highest self-efficacy was reported for Factor 6 “protecting” at 62.2 ± 25.3, compared to males who reported 76.9 ± 21.8 (*P* = .01) for the same factor. Both males and females reported the lowest percentage mean self-efficacy for Factor 5 “funding” at 64.5 ± 24.5 and 47 ± 27.4 (*P* = .01), respectively. This is consistent with previous studies using a variety of measures to assess research self-efficacy among physician-scientists,^22^ biomedical scientists,^34^ undergraduates,^35^ lecturers,^36^ and university faculty.^37^ On the other hand, the only other study of research self-efficacy to have been conducted in Qatar found gender was not an influencing factor to clinical research self-efficacy among primary care physicians employed throughout Qatar's primary health care centers.^16^ There are other studies that suggest gender is not a determinant in many settings or groups.^38^ The observed difference between male and female self-efficacy in this study may reflect specific features in the research environment that could be particular to HMC, such as experience or training, or the more general tendency for men to overestimate and for women to underestimate their efficacy in various tasks.^22^ Other possible confounding factors include fear of “stereotype threat,” which implies that when women are in environments that perpetuate a certain gender-specific stereotype, such as men are better in clinical research, women might be less confident and less likely to engage in clinical research for the fear of being judged as less feminine.^39^ Additionally, other scholars have found that gender differences in self-efficacy can be nullified if previous achievements were accounted for.^38,39^

Level of education was also found to have a strong positive correlation with research self-efficacy for all factors. In our results, participants with MD and PhD degrees reported higher percentage mean self-efficacy than those holding graduate degrees. The highest percentage mean self-efficacy reported by PhD degree holders was for Factor 2 “reporting, interpreting and presenting” at 87.4 ± 14 followed by Factor 3 “conceptualizing and collaborating” at 84.4 ± 14.4. Again, the lowest reported percentage mean self-efficacy for participants holding different degrees was for Factor 5 “funding.” This coincides with what had been observed elsewhere,^31^ with similar increases in all factors (or “domains”) assessed. This also aligns with the findings of another study of MD-PhD students that concluded research self-efficacy increased throughout the course of study progression,^23^ suggesting a likely synergy between degree level and experience within research-oriented degrees. Possible confounding factors that have not been accounted for in this study include clinical research training methods during study programs,^23^ grade point average, and age.^40^ Several authors have explored possible reasons that could lower research self-efficacy among PhD holders, such factors include, attrition in physician-scientist workforces, administrative hurdles, lack of research-protected time, and subjects’ recruitment difficulties.^23,41^

Seniority of position also exhibited a noteworthy trend with statistically significant correlation for all factors except factors 1 and 2. Residents reported the lowest percentage mean self-efficacy for all CRAI factors, compared to senior consultants who reported the highest percentage mean self-efficacy for almost all factors. All participants in various positions reported lowest percentage mean self-efficacy for Factor 5 “funding.” This is consistent with observations elsewhere that clinical positions with supervisory roles may be correlated with higher research self-efficacy.^31^ Indeed, individuals in more senior positions may tend to report higher levels of research self-efficacy simply because they have accumulated greater research experience. Increased research self-efficacy in medicine has also been reported to correlate with a number of publications,^42^ contribution to developing clinical practice guidelines,^16^ and years of experience.^31^

Previous studies have shown a positive effect of research training on self-efficacy.^16,43^ However, respondents in the current study who had received training reported significantly higher levels of self-efficacy in only 2 out of 6 factors compared to those without training. Examined more closely, this effect appears to be mainly due to training received from outside HMC. Moreover, there was no difference in reported self-efficacy between respondents who had or had not received training at HMC, whereas research self-efficacy increased in 3 out of 6 factors among those who had received external training. This difference is perhaps consistent with the correlation between reported self-efficacy and education level, where the associated research training would likely have been received outside HMC. In addition, Bougmiza et al^16^ found training in research methodology was a factor in increasing research self-efficacy among primary care physicians in Qatar, indicating that effective training is available to health care professionals in Qatar.

Perhaps consistent with the lack of impact from internal training is the lack of correlation between research self-efficacy and duration of employment at HMC. The contrasting positive impact of seniority of position on 4 self-efficacy factors suggests that senior clinical researchers had received much or all of their beneficial research experience from outside HMC, possibly prior to employment there. It is important to note here that the roles of clinical research professionals have increased in complexity over time and only until recently; clinical research training started to progress positively on a global scale and meet the evolving requirements of the clinical research enterprise. This improvement was the result of international efforts such as the Joint Task Force (JTF) for Clinical Trial Competency.^31,44^ Other attributes like nationality and departmental affiliation, which were determined to have no impact on research self-efficacy, are consistent with prior findings that examined nationality and specialty as factors for clinical research efficacy among primary care physicians.^16^

### Limitations

There are several limitations to this study. First, the return rate for the survey (13.3%) was very low, which in turn, may allow for slight differences in factors to be undetected. However, the respondents represented all categories of researchers at HMC in terms of position, seniority, education, and specialties. This small sample size might have influenced our interpretation of various factors (gender and educational level) as confounding factors for self-efficacy scores. Second, as the study is cross-sectional, it is possible to establish correlations, but not cause and effect between research self-efficacy and the factors investigated here. Third, while we have tried to draw comparisons between our results and other studies, a variety of measures of self-efficacy are used, and many studies in the biomedical field focus solely on doctoral students rather than professional physicians and researchers.^45^ Fourth, multiple entries originating from computers with the same IP address were retained to account for shared computer usage, which is a widespread practice across HMC. Fifth, self-reporting relies on honest and accurate answers from respondents, which cannot always be assured.^46^ Finally, the CRAI-12 lacks measurable, defined levels of competencies. Having clear guidelines on what each segment would present may aid in categorizing clinical research self-efficacy in future studies that might be using this tool. For example, a possible proposed scheme for the scoring can be divided as follows: fundamental self-efficacy (1-3), skilled (4-6), and advanced (7-10).

### Recommendations

The limited impact of internal training and length of employment on research self-efficacy suggests an opportunity for HMC to enhance or reevaluate its research training initiatives in view of the local priorities and recently defined JTF clinical research core competencies framework or other similar internationally applicable modules with special focus on grantsmanship. External training seems to have been more effective than internal, therefore, a study of external programs might provide insights into developing more effective internal programs, or at least identify which external programs are most beneficial for researchers to attend. Another suggestion would be to promote the pursuit of specialized higher degrees by research staff, as level of education was found to have had a powerful influence on research self-efficacy. The lower levels of research self-efficacy among female researchers may require further investigation, especially as it is not clear that this is the case elsewhere in Qatar. Work and training environments may be experienced differently by men and women and have differing effects on their self-efficacy.^47^ Efforts to provide training for clinical researchers at HMC should consider a variety of approaches, with an awareness of those that may be preferred by women, such as the presence of more female role models and mentors, or the adoption of mentoring models of women, who may face greater challenges balancing work and home demands.^48^

## Conclusions

This research sheds light on the determinants impacting research self-efficacy among researchers at HMC. The study revealed that, in general, HMC researchers demonstrate high clinical research self-assessed efficacy across various factors of the CRAI tool, except for their ability to secure funding, where respondents reported lower scores. Additionally, gender and level of education positively affected research self-efficacy with statistical significance observed across all CRAI factors. A significant finding of this study is the positive impact of training on research self-efficacy. Receiving clinical research training, whether inside or outside HMC, was associated with improved self-reported clinical research efficacy. It is worth noting that the effect was more pronounced for training received outside HMC. While a trend was observed, statistical significance was achieved for specific factors within the CRAI. In all, this study provides insights as to the levels of clinical research self-efficacy that HMC researchers possess, and how HMC could work to improve those levels to develop a clinical research workforce that is more likely to engage in professional clinical research practice.

## Supplemental Material

sj-docx-1-mde-10.1177_23821205241233425 - Supplemental material for An Assessment of Clinical Research Self-Efficacy among Researchers at the Largest Healthcare Institute in Qatar: Recommendations and Future Actions Supplemental material, sj-docx-1-mde-10.1177_23821205241233425 for An Assessment of Clinical Research Self-Efficacy among Researchers at the Largest Healthcare Institute in Qatar: Recommendations and Future Actions by Seba Qussini, Saad Shahbal, Ross MacDonald, Samer Hammoudeh, Zeina Al-Ghoul and Kris Diericks in Journal of Medical Education and Curricular Development

sj-docx-2-mde-10.1177_23821205241233425 - Supplemental material for An Assessment of Clinical Research Self-Efficacy among Researchers at the Largest Healthcare Institute in Qatar: Recommendations and Future Actions Supplemental material, sj-docx-2-mde-10.1177_23821205241233425 for An Assessment of Clinical Research Self-Efficacy among Researchers at the Largest Healthcare Institute in Qatar: Recommendations and Future Actions by Seba Qussini, Saad Shahbal, Ross MacDonald, Samer Hammoudeh, Zeina Al-Ghoul and Kris Diericks in Journal of Medical Education and Curricular Development
