# Supplementary material for: An Assessment of Clinical Research Self-Efficacy among Researchers at the Largest Healthcare Institute in Qatar: Recommendations and Future Actions
Source: J Med Educ Curric Dev. 2024 Jun 7;11:23821205241233425. doi: 10.1177/23821205241233425 (PMC11162134; doi:10.1177/23821205241233425)
Supplement: sj-docx-2-mde-10.1177_23821205241233425 - Supplemental material for An Assessment of Clinical Research Self-Efficacy among Researchers at the Largest Healthcare Institute in Qatar: Recommendations and Future Actions [file sj-docx-2-mde-10.1177_23821205241233425.docx]

| **Gender** | Male |
| --- | --- |
|  | Female |
| **Nationality** |  |
|  |  |
| **Position** | Resident |
|  | Fellow |
|  | Specialist |
|  | Associate Consultant |
|  | Consultant |
|  | Senior Consultant |
|  | Other (Specify) |
| **Degree** | Bachelors |
|  | MSc |
|  | MD |
|  | PhD |
| **Years at HMC** |  |
|  |  |
| **Department** | Medicine |
|  | Surgery |
|  | Pharmacy |
|  | Nursing |
|  | Other (Specify) |
| **Research training at HMC?** | No |
|  | Yes (specify) |
| **Research training outside HMC?** | No |
|  | Yes (specify) |

| **CRAI ITEMS** | |
| --- | --- |
| **Designing and collecting** | |
| Design the best data analysis strategy for your study (analysis) | \| 0 \| 1 \| 2 \| 3 \| 4 \| 5 \| 6 \| 7 \| 8 \| 9 \| 10 \| \| --- \| --- \| --- \| --- \| --- \| --- \| --- \| --- \| --- \| --- \| --- \|   Zero confidence Very Confident |
| Determine an adequate number of subjects for your research project (number of subjects) | \| 0 \| 1 \| 2 \| 3 \| 4 \| 5 \| 6 \| 7 \| 8 \| 9 \| 10 \| \| --- \| --- \| --- \| --- \| --- \| --- \| --- \| --- \| --- \| --- \| --- \|   Zero confidence Very Confident |
| **Reporting, interpreting, and presenting** | |
| Write the results section of a research paper that clearly summarizes and describes the results, free of interpretative comments (results) | \| 0 \| 1 \| 2 \| 3 \| 4 \| 5 \| 6 \| 7 \| 8 \| 9 \| 10 \| \| --- \| --- \| --- \| --- \| --- \| --- \| --- \| --- \| --- \| --- \| --- \|   Zero confidence Very Confident |
| Write a discussion section for a research paper that articulates the importance of your findings relative to other studies in the field (discussion) | \| 0 \| 1 \| 2 \| 3 \| 4 \| 5 \| 6 \| 7 \| 8 \| 9 \| 10 \| \| --- \| --- \| --- \| --- \| --- \| --- \| --- \| --- \| --- \| --- \| --- \|   Zero confidence Very Confident |
| **Conceptualizing and collaborating** | |
| Select a suitable topic area for study (topic) | \| 0 \| 1 \| 2 \| 3 \| 4 \| 5 \| 6 \| 7 \| 8 \| 9 \| 10 \| \| --- \| --- \| --- \| --- \| --- \| --- \| --- \| --- \| --- \| --- \| --- \|   Zero confidence Very Confident |
| Identify faculty collaborators from within and outside the discipline who can offer guidance to the project (collaborators) | \| 0 \| 1 \| 2 \| 3 \| 4 \| 5 \| 6 \| 7 \| 8 \| 9 \| 10 \| \| --- \| --- \| --- \| --- \| --- \| --- \| --- \| --- \| --- \| --- \| --- \|   Zero confidence Very Confident |
| **Planning** | |
| Set expectations and communicate them to project staff (expectations) | \| 0 \| 1 \| 2 \| 3 \| 4 \| 5 \| 6 \| 7 \| 8 \| 9 \| 10 \| \| --- \| --- \| --- \| --- \| --- \| --- \| --- \| --- \| --- \| --- \| --- \|   Zero confidence Very Confident |
| Ask staff to leave the project team when necessary (staff) | \| 0 \| 1 \| 2 \| 3 \| 4 \| 5 \| 6 \| 7 \| 8 \| 9 \| 10 \| \| --- \| --- \| --- \| --- \| --- \| --- \| --- \| --- \| --- \| --- \| --- \|   Zero confidence Very Confident |
| **Funding** | |
| Describe the proposal review and award process for a major funding agency, such as the National Institutes of Health, National Science Foundation, or other foundation (funding) | \| 0 \| 1 \| 2 \| 3 \| 4 \| 5 \| 6 \| 7 \| 8 \| 9 \| 10 \| \| --- \| --- \| --- \| --- \| --- \| --- \| --- \| --- \| --- \| --- \| --- \|   Zero confidence Very Confident |
| Locate appropriate forms for a grant application (grant) | \| 0 \| 1 \| 2 \| 3 \| 4 \| 5 \| 6 \| 7 \| 8 \| 9 \| 10 \| \| --- \| --- \| --- \| --- \| --- \| --- \| --- \| --- \| --- \| --- \| --- \|   Zero confidence Very Confident |
| **Protecting** | |
| Describe ethical concerns with the use of placebos in clinical research (ethics) | \| 0 \| 1 \| 2 \| 3 \| 4 \| 5 \| 6 \| 7 \| 8 \| 9 \| 10 \| \| --- \| --- \| --- \| --- \| --- \| --- \| --- \| --- \| --- \| --- \| --- \|   Zero confidence Very Confident |
| Apply the appropriate process for obtaining informed consent from research subjects (informed consent) | \| 0 \| 1 \| 2 \| 3 \| 4 \| 5 \| 6 \| 7 \| 8 \| 9 \| 10 \| \| --- \| --- \| --- \| --- \| --- \| --- \| --- \| --- \| --- \| --- \| --- \|   Zero confidence Very Confident |
